# Can we cure HTLV-I associated Adult T cell Leukemia Lymphoma?

**DOI:** 10.1186/1742-4690-8-S1-A37

**Published:** 2011-06-06

**Authors:** Ali Bazarbachi, Felipe Suarez, Olivier Hermine

**Affiliations:** 1 Department of Internal Medicine, American University of Beirut, Beirut, Lebanon; 2 CNRS UMR 8603 and Department of Hematology, Necker Hospital, Paris, France

## 

Adult T cell leukemia (ATL) is one of the rare human cancers initiated by a transforming retrovirus, HTLV-I. After many years of controversy, it is now accepted that the viral transactivator protein Tax plays a critical role in initiating the leukemic process, because Tax transgenics develop a disease with striking ATL features. However, its role in the maintenance of the lymphoproliferation remains still a matter of debate. Long-term prognosis of ATL patients remains extremely poor. In acute ATL, well conducted Japanese trials demonstrated that although combinations of chemotherapy improved response rate, they failed to achieve a significant impact on survival. Patients with chronic and smoldering ATL have a better prognosis but long-term survival is poor when these patients are managed with a watchful-waiting policy or with chemotherapy. We recently realized a worldwide meta-analysis showing that the combination of zidovudine and interferon-alpha (IFN) is highly effective in the leukemic subtypes of ATL and should be considered as standard first line therapy in that setting. This combination has changed the natural history of the disease through achievement of significantly improved long-term survival in patients with smoldering and chronic ATL as well as patients with acute ATL and wild type p53. ATL lymphoma patients may benefit from initial induction therapy based on aggressive chemotherapy regimen in addition to or followed by antiretroviral therapy with AZT/IFN. In all patients, in order to prevent the occurrence of resistance and relapse, clinical trials assessing bone marrow transplantation additional targeted therapies such as arsenic/IFN combination or monoclonal antibodies, are mandatory after achieving CR. In that sense, we recently reported that the combination of arsenic trioxide, IFN, known to trigger Tax proteolysis in addition to zidovudine, yielded unprecedented response rates in chronic ATL patients, and may prevent relapses in ATL lymphoma responding patients after chemotherapy. To investigate the molecular mechanism of therapeutic action in vivo, we used Tax transgenic mice that develop a disease with striking ATL features. We demonstrate that the combination of arsenic trioxide and IFN cures Tax-driven murine ATLs through selective targeting of leukemia initiating cell (LIC) activity. Importantly, this effect requires proteasome function. Overall, our findings strongly suggest that in this model ATL LICs are addicted to the iral Tax oncoprotein and open the prospect of new strategies to cure ATL.

